# Social determinants of mid-life mental health decline: a life-course analysis from the 1970 British Cohort Study

**DOI:** 10.1093/eurpub/ckag146

**Published:** 2026-08-13

**Authors:** Christoph Henking, Aaron Reeves

**Affiliations:** Department of Social Policy and Intervention, University of Oxford, Oxford, United Kingdom; Department of Sociology, London School of Economics and Political Science, London, United Kingdom

## Abstract

Mental well-being has been shown to follow a U-shaped pattern across the life course in cohorts studied to date, with psychological distress worsening as people enter their forties. The reasons for this decline remain a puzzle. This study examined the social determinants associated with this phenomenon and their relative importance as predictors of mid-life mental health. We used data from the 1970 British Cohort Study, aged 26, 34, and 42 (*N* = 6992, 51.5% female). Mental distress was measured with the nine-item Malaise Inventory. We contrasted a group of those whose mental health declined with those exhibiting stable mental health. Random forest (RF) and logistic regression models explored whether decline was predicted by socio-economic and family factors, physical health, and health behaviours (at ages 26, 34, or birth). Social class at birth (variable importance [VI] = 0.027) and income quintile at 34 (VI = 0.027) were the most important predictors in RF analysis. Regression confirmed that working-class background (odds ratio [OR] = 1.53 [1.27–1.85]) and being in the highest-income quintile at 34 (OR = 0.68 [0.53–0.88]) strongly predicted the mental health decline. The predicted probability of decline was two and a half times higher for those with an unfavourable, compared to a favourable, combination of social determinants (28% vs 11%). Importantly, a working-class background was not associated with mental health decline between ages 26 and 34. Early-life social class and income were among the strongest predictors of mid-life psychological distress. Decline was concentrated among those exposed to economic disadvantage, suggesting that this group should be a particular focus of mid-life mental health prevention and support.

## Introduction

There is strong evidence that mental well-being declines for many people in midlife before increasing again towards older ages in cohorts studied to date, producing a U-shaped pattern over the course of adulthood [1, 2]. Studies using British birth cohorts have consistently identified the period between approximately ages 30 and 45 as a stage during which mental health deteriorates for a substantial proportion of individuals [1–4]. In this study, we focus specifically on this segment of the adult life course: the trajectory from early adulthood into mid-life.

While the reasons for the U-shaped pattern remain unclear [5, 6], there are plausible biological and psychological explanations. A similar decline of well-being over the middle phase of the life course is observable in other primates suggesting this trend may be driven by the biological effects [6]. Psychological explanations suggest people experience more negative emotions and regret in mid-life and then becoming more content towards later adulthood [7, 8].

Alongside these explanations, there might be social determinants of this mid-life decline [9]. We know that mental health is correlated with various dimensions of ‘socio-economic position’, such as social class, gender, and occupational group, as well as ‘intermediary determinants’ such as working conditions, family support, and social relationships [10, 11]. These social determinants can affect mental health throughout the life course by altering material circumstances, exposure to stress, and health behaviours [12, 13]. Given their importance throughout the life course, an important question is whether the distribution of social determinants can help explain why some individuals experience a deterioration in mental health as they approach mid-life whereas others do not.

We argue that these social determinants may be associated with mental health decline because of effect heterogeneity which varies throughout the life course. In other words, the influence of particular social determinants may become stronger as people approach mid-life [6, 14]. For example, individuals experiencing low income and financial hardship are more likely to experience a mid-life decline [14] as well as those with lower education [15]. It could be that income and education loom particularly large in this period as socio-economic situations become more fixed and upward social mobility becomes less likely than in early adulthood [15, 16]. Therefore, health inequalities based on these socio-economics might widen, which could be reflected in the mid-life decline.

Whether the mid-life decline is socially patterned has direct implications for public health and healthcare planning. If mental health deterioration is concentrated among particular socio-economic groups, this would suggest that the mid-life decline is not simply an age-related phenomenon. Identifying which groups are at elevated risk may help inform prevention efforts, service planning, and the targeting of support, in accordance with the principle of proportionate universalism [17, 18].

However, most research focused on modelling the U-Shape pattern of well-being in various contexts and treated factors like gender and socioeconomic status as control variables [4, 5], rather than directly investigating how specific social determinants are associated with the mid-life decline. Therefore, the social factors and mechanisms underlying mid-life mental health remain poorly understood.

In this study, we investigate the mid-life decline in mental health from a social determinants of health perspective.

We aim to (i) describe the socio-economic characteristics of individuals who experience a mid-life decline in mental health, (ii) identify which of the social determinants of mental health have the greatest predictive importance in the random forest (RF) model of the subsequent mid-life decline, and (iii) estimate differences in the predicted probability of mental health decline across population risk profiles. We focus on the period between ages 34 and 42 in the 1970 British Cohort Study, a stage of adulthood that previous research has identified as a key period of increasing psychological distress [1–4].

## Methods

### Study population

The data for this prospective observational study were derived from the 1970 British Cohort Study (BCS 1970). The BCS started with a representative sample of 17 196 individuals born in the UK in 1 week in 1970 [19, 20]. Participants were the same age at every survey wave, with waves happening every 4–8 years. Following previous research, we selected the waves that include the Malaise Inventory to measure psychological distress (ages 26, 34, and 42). Our primary focus is on the change in mental health between the ages of 34 and 42 where the mid-life increase in psychological distress occurs in the BCS 1970 cohort while the prevalence of mental health problems stabilizes between the ages of 42 and 46 [1–3]. We therefore focus our analysis on the specific mental health decline between ages 34 and 42. We included all participants who provided a valid response to the mental health items at ages 34 and 42 (*N* = 6992 participants; 51.5% female, 48.5% male).

### Dependent variable

The dependent variable of the study is ‘decline in mental health'. Firstly, to measure it, we started by assessing mental health using the short version of the Malaise Inventory, which was used at the ages of 26, 34, and 42 to measure psychological distress [1, 20, 21]. The scale uses binary questions to test for different symptoms of psychological distress, forming a sum score for each individual from 0 to 9. Lower values on the scale indicate lower psychological distress, while values over three indicate high distress and risk of depression [3, 22]. The Malaise Inventory has shown good validity compared to other scales and has been frequently used in similar studies concerning population mental health [21, 22]. Secondly, we defined the decline in mental health as an increase in psychological distress on the Malaise scale by at least two points (one standard deviation) between the ages of 34–42. There is no universally established deterioration threshold for the 9-item Malaise Inventory. We selected this threshold to capture a meaningful within-person change in psychological distress, consistent with distribution-based approaches used elsewhere, where one-half standard deviation is a common benchmark for minimally important change [23, 24]. Our threshold therefore exceeds this benchmark and can be considered relatively conservative. All other individuals are included in the ‘stable mental health’ group (see Supplementary Table S1 in Appendix A). Following this definition, 17.8% of individuals (*n *= 1247) were identified as experiencing a decline in mental health. To verify the robustness of results, we also include different definitions of mental health decline in our supplementary analysis (see Supplementary Section B1 in Appendix B).

### Predictors

Predictors in this study were selected from survey waves at birth and at ages 10, 26, and 34 to be prognostic of the change in psychological distress from age 34–42. Some variables were only available at age 34 (see Supplementary Table S1). Variable selection was based on the framework of the Social Determinants of Health by the World Health Organization (WHO) [9]. Here, the social determinants were grouped into *Socio-Economic Position* (social class, education, income, employment status, and sex) and *Intermediary Determinants* (living and working conditions, family, and behavioural factors). This also corresponds to similar public health research based on the WHO framework [11, 25].

As indicators of Socio-Economic Position, we used sex assigned at birth as well as social class at birth. Social class at birth was measured through the father’s occupation following the Register General’s classification [3] with a categorization as ‘low’ (IV partly skilled/V unskilled), ‘medium’ (III skilled nonmanual/manual), and ‘high’ (I professional/II intermediate). Education at age 34 was categorized into ‘high’ (university/higher education), ‘medium’ (completing A-levels), and ‘low’ (GCSE and below). Self-reported income is the total take-home pay in British Pounds and was categorized into 1 = lowest; 5 = highest income quintiles. Employment status was categorized into ‘full-time’, ‘part-time’, ‘unemployed’, ‘looking after home/family’, and ‘sickness/disability’. We could not include a substantive indicator of ethnic group due to the low diversity in terms of ethnic background in the BCS 1970 (94.2% British white participants and 96.2% white overall [19]).

As Intermediary Social Determinants, working conditions were measured as job insecurity, low job satisfaction, and working multiple jobs to make ends meet [8, 26]. Marital status and number of children were included as family factors. Behavioural factors included exercise and smoking (while dangerous alcohol consumption was not measured).

Furthermore, we also included a measure of physical multimorbidity (having at least two medical conditions) as it was shown to be strongly associated with psychological distress [22] and Cognitive ability in childhood (age 10) which is a relevant covariate for developments throughout the life course (see Supplementary Section A1 in Appendix A for more details).

### Statistical analysis

Our research involves exploratory analyses based on a variety of predictors, followed by confirmatory analyses to establish effect sizes. For the purpose of exploration based on many variables, we combine the machine learning algorithm of RF for variable selection with established regression methods which provide effect estimation and confidence intervals [27]. This combination of different analysis methods—descriptive comparisons, RF variable selection, and regression-based effect estimation—helps overcome issues of regression models with a large number of variables such as multicollinearity and overfitting and provides an in-built triangulation of our findings throughout statistical approaches [28].

In the first step (description), the dataset is split into two analysis groups (stable vs decline in mental health), and the distribution of the social determinants at age 34 and 26 in both groups is compared using simple *t*-tests. In the second step (variable selection), the goal is to identify the most predictive variables of being in the group of mental health decline. We use an RF algorithm which is well suited for exploratory analyses with a high number of potential explanatory factors and addressing multicollinearity issues [28]. This technique was used in previous analyses of predicting self-reported health based on several social and genetic determinants [29]. We use the R package ‘party’ and its function ‘cforest’ which produces an ensemble of 1000 prediction trees, tailored to handle different variable types and inter-correlated predictors [27]. This algorithm calculates the variable importance (VI; root mean squared error) for each predictor that indicates which one is the most essential for accurately forecasting mental health outcomes (predicted probability between 0 and 1 for each participant to experience a decline in mental health) across the 1000 computations. Importantly, RF VI should not be interpreted as evidence of causal effects. VI estimates identify predictors that improve model performance but do not establish whether changing a particular determinant would alter the risk of mental health decline [30]. Furthermore, VI measures can be influenced by correlations between predictors and other modelling choices [31]. We also used LASSO regression as an alternative machine learning approach which produced consistent findings (Supplementary Fig. S1 in Appendix B).

In the third (estimation of effect sizes) step, we selected the 12 predictors with the highest VI in step 2. For each of these variables, we construct logistic regression models with a theoretically appropriate set of control variables to reveal effect sizes as odds ratios (ORs) and predicted probabilities of mental health decline depending on each identified predictor and all predictors combined (see Supplementary Section A5 in Appendix A for details on co-variate selection). Finally, we calculate the same estimates for the age shift in mental health from 26 to 34 to analyse if the identified predictors of decline are specific to the age period of 34–42 or are also associated with changes in the previous age period.

All participants who provided a valid answer to the question on mental distress at ages 34 and 42 are included in our analyses (*N* = 6992), and we impute missing data on all other covariates using the R package RF. Details on our handling of missing values can be found in Supplementary Appendix B (Section B3).

## Results

In line with previous research, psychological distress increased from age 34 (*M* = 1.67, standard deviation [SD] = 1.9 on the nine-point Malaise Inventory) to 42 (*M* = 1.86; SD = 2.0) with *t*-tests revealing a significant difference between these values (*P *< .001), while there is no further increase to the age of 46 (*M* = 1.77). Descriptive statistics on all variables are shown in Appendix A (Supplementary Table S1).

### Characteristics of decline vs stable groups (step 1)

First, we divided the sample into those who experienced a decline in mental health (i.e. an increase in psychological distress) and explored group differences based on the different social determinants of health. Table 1 reveals the results for the predictors at age 34 (for age 26, results are shown in Supplementary Table S2, Appendix A). *t*-Tests reveal that the group of declining mental health consists of significantly more individuals from low social class backgrounds compared to the stable group (23.5% vs 17.8%; *t *= 4.23; *P *< .01). It also contains fewer individuals with high income at age 34 (11.4% vs 15.6%; *t *= 4.07; *P *< .01), more unemployed people (2.4% vs 1.5%; *t *= 1.97; *P *= .049), and taking care of home/family (12.0 vs 9.4%; *t *= 2.56; *P *= .01). Those who declined in mental health also less frequently reported regular exercise (77.1% vs 80.5%; *t *= 2.66; *P *< .01).

**Table 1.** Description of the groups of stable mental health and mental health decline

Social determinants (at age 34)	Share in group of stable mental health	Share in group of mental health decline	Difference	*P*-value (*t*-test)
Sex: female	53.4%	55.5%	2.1%	.179
Social class				
Low birth social class	17.8%	23.5%	5.7%	<.001
High birth social class	21.9%	20.1%	−1.8%	.173
Education				
University education	36.5%	33.6%	−2.9%	.051
Income and employment				
Income quintile 5 (highest)	15.6%	11.4%	−4.2%	<.001
Income quintile 1 (lowest)	13.3%	15.0%	1.7%	.129
				
Working part-time	16.2%	15.5%	−0.7%	.557
Unemployed	1.5%	2.4%	0.9%	.049
Having a disability	2.1%	1.8%	−0.3%	.489
Looking after home/family	9.4%	12.0%	2.6%	.010
Working conditions				
High job security	93.1%	92.4%	−0.7%	.445
High job satisfaction	80.6%	80.5%	−0.1%	.922
Working multiple jobs	6.3%	6.7%	0.5%	.588
Family factors				
Having children	59.4%	60.4%	1.0%	.531
Being divorced	3.4%	3.4%	0.1%	.927
Being married	56.4%	54.0%	−2.4%	.125
Physical and behavioural factors				
Physical multimorbidity	7.9%	9.3%	1.4%	.128
Regular exercise	80.5%	77.1%	−3.5%	.008
Weekly smoking	29.0%	30.7%	1.8%	.220

Decline in mental health is defined as an increase in psychological distress on the Malaise Scale by at least two points (=1 SD) from the age of 34–42; all other individuals are included in the “stable mental health” group.

### Identifying main predictors of mid-life decline (step 2)

**Figure 1.**
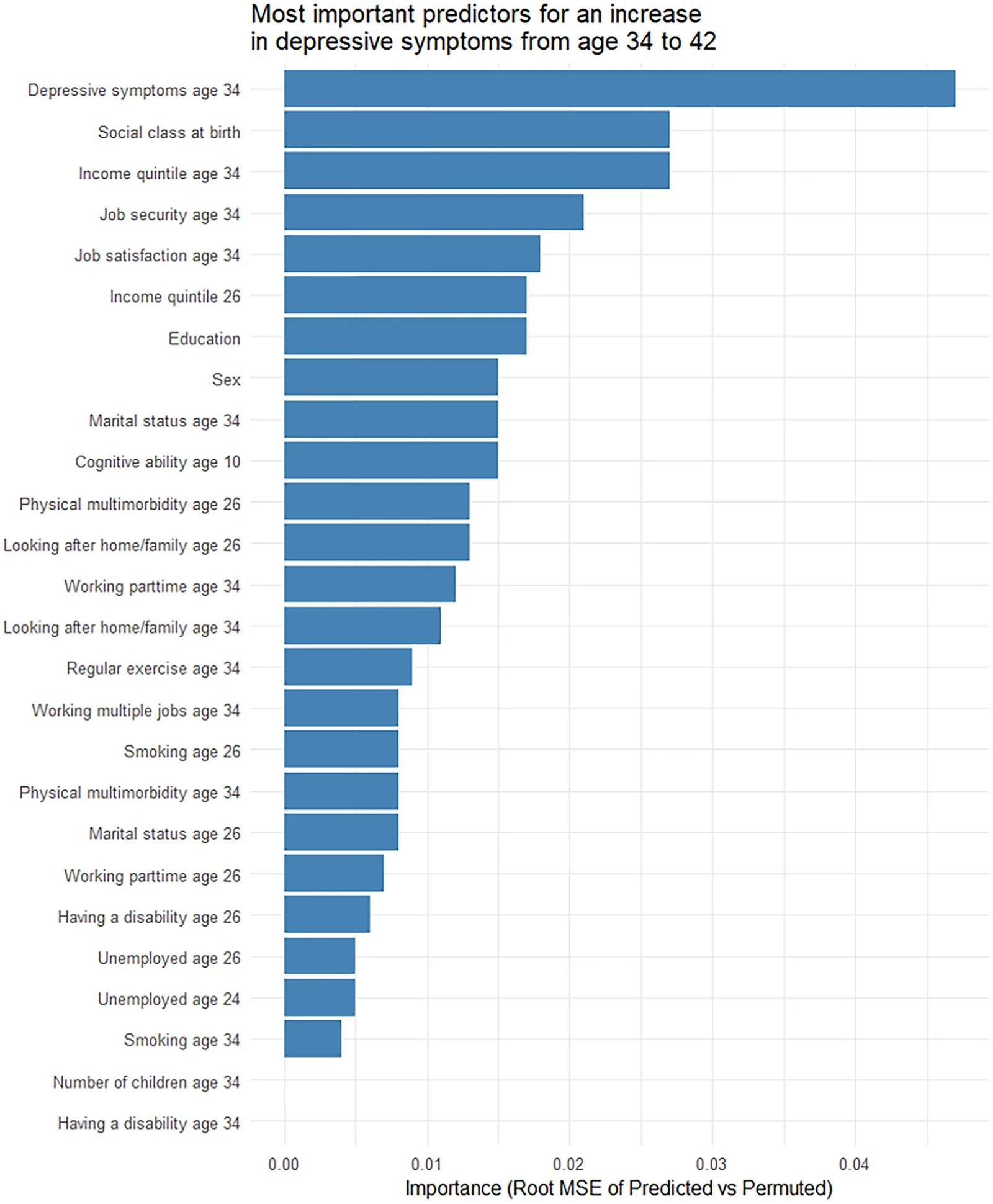
Variable Importance (VI) based on a Random Forest (RF) to predict mid-life decline in mental health.

Next, an RF algorithm was used to identify which social determinants had the greatest predictive importance for decline in mid-life mental health. The result of this algorithm is shown in Fig. 1 as a VI plot. The RF’s VI (RMSE) shows which variables were the most important for the algorithm to improve prediction accuracy. Besides the ‘baseline value’ of psychological distress score at age 34 (strongest predictor for the mental health decline; VI = 0.047), the four strongest predictors were social class at birth (VI = 0.027), income quintile at age 34 (VI = 0.027), job security (VI = 0.021), and job satisfaction (VI = 0.018). Income at age 26, education, sex and physical multimorbidity, marital status, cognitive ability, as well as employment status (working part-time and taking care of home) were also included in the 12 main predictors. This indicates that a broad distribution of social determinants was associated with mid-life mental health changes.

### Predicted probabilities of different risk profiles (step 3)

We selected the 12 predictors with the strongest predictive importance identified by the RF model (beyond psychological distress at age 34) and used logistic regression models to determine the estimate of effect sizes and differences in predicted probabilities of mental health decline associated with each social determinant. If a predictor was chosen at both ages 26 and 34, we used the stronger one with higher VI in our regression model. For each of the models, we selected relevant covariates as outlined in Appendix A (section A5). As shown in Table 2 (model M1), being born into a low social class family was associated with an increased probability of an increase in psychological distress (OR = 1.53, confidence interval [CI]: 1.27–1.85) while being male is linked to a lower probability (OR = 0.87, CI: 0.77–0.98). Being in the highest income quintile at age 34 (see M3) was associated with a lower probability (OR = 0.68, CI: 0.53–0.88). Education (M2), employment (M4), and working conditions (M5; job security and satisfaction) are associated in the expected direction but are not statistically significant (e.g. association of high job security with probability of mental health decline is OR = 0.90, CI: 0.69–1.18). As for family factors (M6), neither being married (OR = 0.94, CI: 0.67–1.34) nor the number of children was significantly associated with experiencing a mental health decline. Finally, having physical multimorbidity at age 26 (M7) was associated with a significantly increased probability of mental health decline (OR = 1.26, CI: 1.06–1.49). Overall, social class at birth, sex, income at age 34, and physical multimorbidity at age 26 were identified as significant predictors of the mid-life mental health decline.

**Table 2.** Logistic regression; outcome: the likelihood of experiencing a mid-life decline in mental health from age 34–42 (OR)

	M1	M2	M3	M4	M5	M6	M7
	Social class	Education	Income	Employment status	Work conditions	Family factors	Physical health
Social class							
Low social class at birth: (ref = high)	1.53	1.42	1.40	1.40	1.38	1.41	1.42
	[1.27–1.85]	[1.16–1.73]	[1.15–1.72]	[1.15–1.71]	[1.13–1.69]	[1.16–1.73]	[1.16–1.74]
Medium social class at birth: (ref = high)	1.09	1.04	1.04	1.04	1.03	1.04	1.04
	[0.93–1.28]	[0.88–1.23]	[0.88–1.23]	[0.88–1.23]	[0.88–1.22]	[0.88–1.23]	[0.88–1.23]
Income and employment							
Income quintile 2 (ref = q1)			0.87	0.88	0.87		
			[0.69–1.10]	[0.69–1.10]	[0.69–1.10]		
Income quintile 3 (ref = q1)			1.02	1.01	1.01		
			[0.81–1.27]	[0.81–1.27]	[0.81–1.27]		
Income quintile 4 (ref = q1)			0.77	0.77	0.76		
			[0.60–0.98]	[0.60–0.97]	[0.60–0.97]		
Income quintile 5 (ref = q1)			0.68	0.67	0.67		
			[0.53–0.88]	[0.52–0.87]	[0.52–0.87]		
Looking after home/family (ref = all other)				1.15			
				[0.90–1.47]			
Working parttime				0.87			
				[0.70–1.08]			
Working conditions							
Job satisfaction high (ref = low)					0.83		
					[0.65–1.07]		
Job security high (ref = low)					0.90		
					[0.69–1.18]		
Family factors							
Being married at age 34 (ref = unmarried)						0.94	
						[0.67–1.34]	
Having no children aged 34 (ref = 1 child)						0.91	
						[0.77–1.08]	
Having 2–3 children (ref = 1 child)						0.94	
						[0.80–1.11]	
Having four or more children						0.93	
						[0.55–1.51]	
Behavioural factors and physical health							
Having physical multimorbidity aged 26 (ref = no)							1.26
							[1.06–1.49]
Covariates							
Mental health age 34	0.88	0.88	0.87	0.87	0.87	0.88	0.87
	[0.85–0.92]	[0.85–0.91]	[0.83–0.91]	[0.84–0.91]	[0.83–0.90]	[0.84–0.91]	[0.84–0.91]
Sex (being male)	0.87	0.86	0.92	0.90	0.95	0.86	0.88
	[0.77–0.98]	[0.76–0.97]	[0.81–1.05]	[0.78–1.05]	[0.83–1.09]	[0.75–0.97]	[0.77–0.99]
University education aged 34 (ref = low education)		0.90	0.99	1.00	0.99	0.91	0.91
		[0.78–1.05]	[0.85–1.16]	[0.85–1.16]	[0.85–1.16]	[0.78–1.05]	[0.78–1.05]
Cognitive ability aged 10		0.99	0.98	0.99	0.99	0.99	0.99
		[0.98–1.00]	[0.97–1.01]	[0.98–1.01]	[0.98–1.01]	[0.98–1.00]	[0.97–1.00]

95% CIs are given within brackets. *N* = 6992 for all analyses.

Based on these four significant predictors and models (M1: social class and sex, M3: income, M7: physical multimorbidity), we calculate the predicted probability of a mental health decline for three scenarios each: one with the most positive level of the risk factor (e.g. income quintile = 5), one with the most adverse level (e.g. income quintile = 1), and one with the mean in the observed study population. All other control variables in the model were set to the mean in the study population.

**Figure 2.**
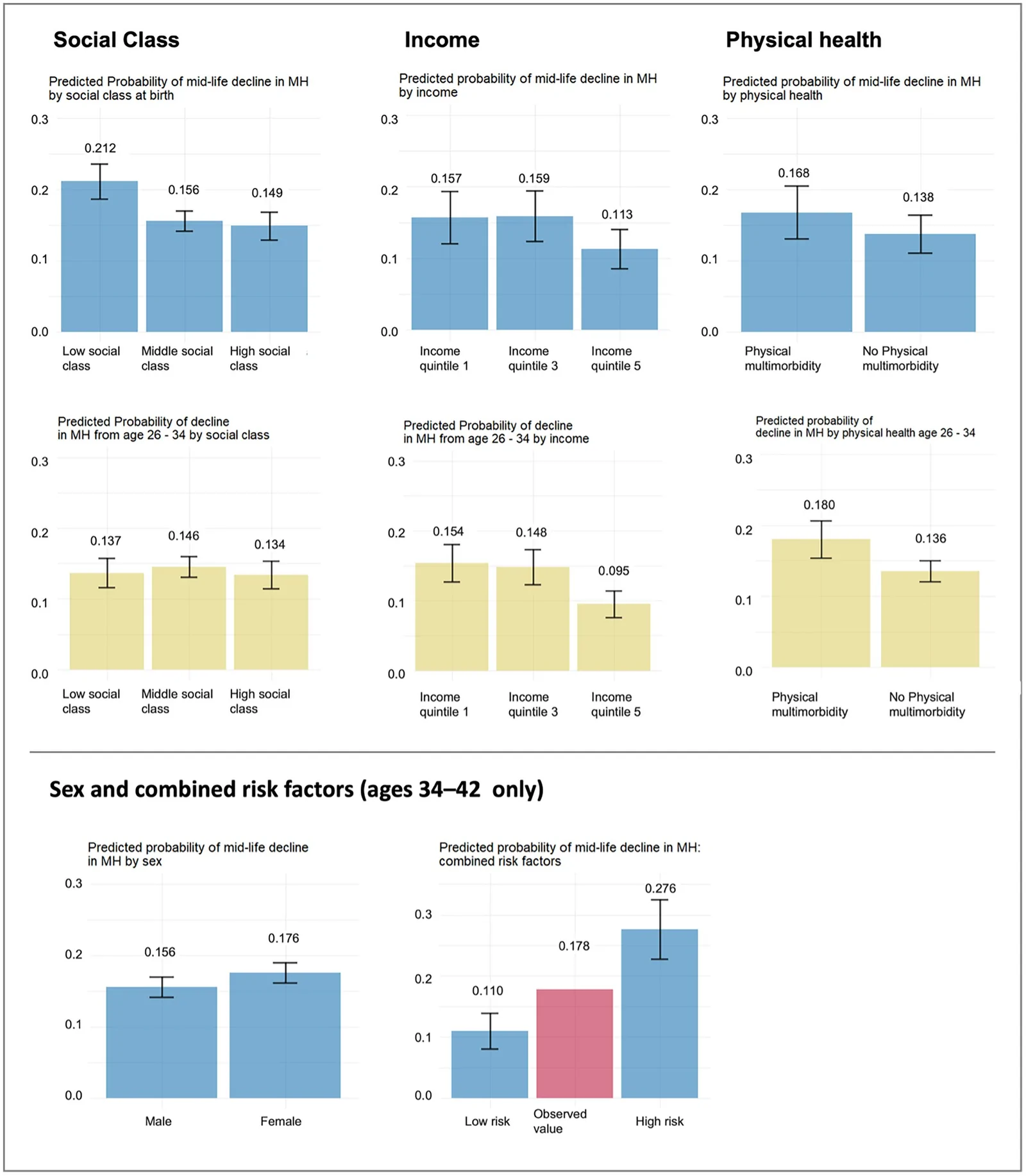
Predicted probabilities based on the logistic regression models shown in Table 2. Blue bars show predicted probabilities for the 34–42 age period; yellow bars show predicted probabilities for the 26–34 age period. In the combined risk factors panel, the pink bar shows the observed prevalence of mental health decline in the study sample, compared with model-based predicted probabilities for low- and high-risk profiles (blue bars).

For low social class at birth (top left in Table 2), the predicted probability of mid-life mental health decline was 0.212 (CI: 0.187–0.236), but 0.156 (CI: 0.142–0.171) for medium social class and 0.149 (CI: 0.129–0.168) for high social class. For income, being in quintiles 1 and 3 had similar probabilities (0.159 CI: 0.124–0.194 for quintile 3), but being in the income quintile 5 at age 34 was associated with a lower probability of 0.113 (CI: 0.085–0.141). The ‘all risk factors combined’ analysis (see Fig. 2) revealed that a favourable value of all risk factors (i.e. high social class, income quintile 5, being male, and not having physical multimorbidity) corresponds to a model-based predicted probability of mental health decline of 0.11 (CI: 0.081–0.139), whereas an adverse combination of factors corresponds to a predicted probability of 0.28 (CI: 0.227–0.324). This compares with an observed prevalence of 17.8% in the study population.

Finally, we explored whether the identified variables were also predictive of changes in mental health in the earlier life period. This allows us to examine whether the identified predictors are unique to the changes during mid-life or are also relevant during the earlier life period. Therefore, we performed equivalent logistic regression analyses and predicted probabilities for social class, income, and physical multimorbidity on the decline in mental health from the ages of 26–34. Sex and the combined risk factor model were examined only for the 34–42 period. The predicted probabilities are shown in Fig. 2 (yellow bars). The results showed that income and physical multimorbidity at age 26 were associated with mental health decline from ages 26–34, as they were for the 34–42 period.

However, the association with social class at birth is different. Being born into a low social class did not predict a mental health decline from age 26–34 (low social class predicted *Pr* = .137; CI: 0.116–0.158; medium social class *Pr* = .146 CI: 0.131–0.160; high social class *Pr* = .134; CI: 0.114–0.154), which indicates that the association of social class with mental health varies as people approach mid-life. The full regression analysis (equivalent to Table 2) can be found in Supplementary Table S3 in Appendix B.

## Discussion

Our study investigated which social determinants were most strongly associated with increases in psychological distress during mid-life. We find that social class at birth, income, physical multimorbidity, and sex are the strongest predictors of mental health decline and are thus associated with social inequalities in mental health during this life stage. In the observed study population, 18% of participants experienced a decline in mental health, whereas the model-based predicted probability ranged from 11% among individuals with a favourable risk profile to 28% among those with an adverse risk profile.

Our findings confirm income as a crucial determinant of health as it both signals social status and directly enables access to resources [3, 14]. Our research is also in line with findings that women have a higher risk for a mental health decline in mid-life and the bidirectional relationship between physical health and mental health [3, 22]. However, while working conditions (such as job security and satisfaction) were predictors of mental health decline in our machine learning model, they did not remain relevant when controlling for income and other confounders. Similarly, family factors such as marital status or having children at age 34 were not associated with who experiences a mid-life decline in the following years. Hence, the majority of identified social determinants (social class at birth, gender, and income quintile) are not novel to mid-life and remain relatively stable over the observed period. On the other hand, many factors associated with frequent life events in mid-life (e.g. getting married, having children, working part-time) did not appear to drive the change in population or inequalities in mid-life decline. Together, these results suggest that *effect heterogeneity* is critical for understanding mid-life decline. In this view, certain pre-existing life circumstances start to have a stronger impact on mental health during mid-life, and it does not seem to be the case that this decline is driven by specific life events becoming more common [14]. This could particularly be the case for social class at birth which was identified as a latent risk factor and which has a distinct association with mental health in the age period between 34 and 42, (but not the previous age period between 26 and 34) [15, 16].

This raises another question: why might early-life disadvantage become more strongly associated with mental health during mid-life? Although our data cannot directly test these mechanisms, several explanations are plausible. One possibility is that opportunities for upward social mobility become more limited as individuals move through adulthood. Early adulthood may offer some opportunity to move out of socio-economic disadvantage, but these opportunities diminish over time as access to further education and training or, other routes to changing economic circumstances become more limited [32]. This could be associated with poorer mental health among those who were not upwardly mobile, consistent with evidence that moving out of hardship later in life, compared with early adulthood, is more difficult and entails fewer health benefits [33, 34].

A second possibility relates to the accumulation of social and economic stressors across generations. Through a mechanism of intergenerational transmission, parents from lower socio-economic backgrounds are more likely to experience poorer health themselves [35, 36], which may increase the financial and caregiving demands placed on their adult children during mid-life, alongside the demands of raising their own families [37].

This combination of additional demands could increase vulnerability to psychological distress during the mid-life period, consistent with an effect-heterogeneity account.

These findings have implications for policy and practice. First, they reinforce the case for reducing childhood poverty [38], given that early-life financial insecurity was associated with mental health problems persisting into mid-life. Second, they suggest that mid-life mental health prevention and support may be most effective if scaled to need. Universal mental health prevention should set mid-life as a priority for all—given the observed decline in this age period—but with particular emphasis on those experiencing financial hardship in childhood or adulthood [17, 18]. This could be achieved through screening and outreach integrated into services already in contact with this group, such as primary care, employment support, or financial advice services.

Several limitations need to be noted. First, our study population lacks ethnic diversity [9]. Our findings need to be verified for a more ethnically diverse population. Second, we only include social determinants until age 34 to be prognostic of the subsequent change in mid-life. Therefore, we do not investigate direct changes between ages 34 and 42, which would be needed to test different theoretical assumptions (e.g. heterogeneity vs life events perspectives). Third, RF VI reflects predictive relevance, not causal influence: it identifies predictors that improve model performance but does not establish whether changing a particular determinant would alter the risk of mental health decline [34]. To mitigate this limitation, we compared findings across descriptive analyses, RF models, logistic regressions, and robustness analyses using alternative outcome definitions. The consistency of findings across these complementary approaches aims to increase confidence in our findings [27, 28]. Fourth, our empirical focus is restricted to changes from early adulthood into mid-life. We therefore cannot assess how our findings relate to mental health problems in adolescence or later older age [39]. Fifth, the U-shaped pattern in well-being observed across earlier cohorts may not generalize to younger cohorts, where rising rates of poor mental health in adolescence and early adulthood could alter this trajectory [40].

Overall, we find that social class at birth, income, physical multimorbidity, and sex were the strongest predictors of mid-life mental health decline in the British Cohort Study born in 1970. The findings suggest that the mid-life decline is socially patterned and concentrated among individuals exposed to social and economic disadvantage. This points to mid-life as a key period for mental health support that is scaled to need and targeted at those who experienced early-life disadvantage, alongside continued efforts to reduce childhood poverty.

## Supplementary Material

ckag146_Supplementary_Data

## Data Availability

The dataset used for this analysis—the 1970 British Cohort Study—is accessible via the UK data service at https://datacatalogue.ukdataservice.ac.uk/series/series/200001#abstract. Key points Mental health typically worsens in mid-life, but the social determinants of this decline are poorly understood. Using the 1970 British Cohort Study, we show that early-life social class and mid-life income are the strongest predictors. Working-class origin increases the risk of mid-life mental health decline, while higher income at 34 is protective. The influence of early disadvantage appears specific to mid-life, not earlier adulthood. Addressing both childhood disadvantage and mid-life economic insecurity may help prevent mental health decline.
