# Supplementary material for: Social determinants of mid-life mental health decline: a life-course analysis from the 1970 British Cohort Study
Source: Eur J Public Health. 2026 Aug 13;36(5):ckag146. doi: 10.1093/eurpub/ckag146 (PMC13472683; doi:10.1093/eurpub/ckag146)
Supplement: Supplementary file 1 [file ckag146_supplementary_data.zip › ejph-2025-09-om-0797-File004.docx]

**Appendix A**

Appendix A has the purpose of providing additional information on the variable descriptions and descriptive analyses. Section A1 provides additional information on the predictors used in the main analysis. Section A2 provides the descriptive statistics for the different waves of all variables used in the dataset. Section A3 provides additional analysis of Research Question 1 (“Characteristics of decline vs. stable groups”), by providing an overview of the predictors at age 26. Section A4 provides more details on the RandomForest algorithm used in the main text. Finally, section A5 provides more details on the reasoning for the selection of co-variation in the regression analysis.

**A1: Additional information on the predictors**

Job security was measured using the question “Would you say your current job is... “very secure”, “fairly secure” (both classified as “secure job”), “not very secure” (“low job security”). Having multiple jobs was “How many jobs do you have?” and classified as “multiple jobs” or “single job”. Job satisfaction was measured using the item “how satisfied or dissatisfied are you with your present job overall” and categorised into “high” (Very satisfied or satisfied) “medium” (Neither satisfied nor dissatisfied) or “low” (Very dissatisfied or dissatisfied). As family factors, following ^9^, marital status was categorised into “never married”, “married”, “divorced” and “widowed”. Having children was measured as “number of cohort member's natural children in household”. As behavioural factors, smoking was categorised into “current smokers” and “non-smokers”. Exercise was measured whether participants exercise regularly (at least once a month) which classified as “yes” or “no”.

Having physical multimorbidity was defined as self-reporting at least two chronic medical conditions following previous research^20^. These conditions include: asthma; backache; bladder or kidney conditions; cancer; cardiovascular conditions; convulsions or epilepsy; diabetes; hearing conditions; migraine as well as stomach, bowel, or gall conditions^20^. Finally, cognitive ability in childhood (at age 10) was measured using the “British Ability Scales” (BAS), examining Inductive, non-verbal reasoning (28 items; 1 point for each question) ^30^, which is a relevant covariate for developments in mid-life.

**A2: Descriptive statistics**

Table S1 reveals the descriptive statistics for this paper. The variables are shown for the different waves 26 and 34. For the age of 42, only the variable “Psychological distress” is shown as we only included predictor variables up to the age of 34.

| **Table S1.** Descriptive statistics by survey wave (before imputation of missing values). | | | | |
| --- | --- | --- | --- | --- |
| **General sample characteristics** | |  | | |
| Social class at birth | High | 1406 (21.6%) |  |  |
|  | Medium | 3860 (59.3%) |  |  |
|  | Low | 1246 (19.1%) |  |  |
| Cognitive ability age 10 | mean (sd) | 16.42 (5.16) |  |  |
| Sex | Male | 3232 (46.2%) |  |  |
|  | Female | 3760 (53.8%) |  |  |
| **Variables in adulthood** |  | **Age 26** | **Age 34** | **Age 42** |
| Psychological distress | Mean Malaise  score (SD) | 1.7 (1.7) | 1.6 (1.9) | 1.8 (2) |
| Education | University degree |  | 2516 (36.0%) |  |
|  | Completed A-levels | n.a. | 674 (9.6%) |  |
|  | GCSE and below |  | 3797 (54.3%) |  |
| Income is the total take-home pay in British Pounds | Mean (sd) | 1,774 (4,739) | 2,109 (10,036) |  |
|  | Working parttime | 400 (7.6%) | 1121 (16.0%) |  |
|  | Unemployed | 162 (3.1%) | 116 (1.7%) |  |
|  | Having a disability | 70 (1.3%) | 140 (2.0%) |  |
|  | Looking after home/family | 509 (9.7%) | 689 (9.9%) |  |
| Job security | High | n.a. | 5460 (78.3%) |  |
|  | Low |  | 411 (5.9%) |  |
|  | Not applicable |  | 1104 (15.8%) |  |
| Job satisfaction | High | n.a. | 4745 (67.9%) |  |
|  | Medium |  | 644 (9.2%) |  |
|  | Low |  | 499 (7.1%) |  |
|  | Not applicable |  | 1104 (15.8%) |  |
| Working multiple jobs | Yes | n.a. | 5514 (93.6%) |  |
|  | No |  | 374 (6.4%) |  |
| Marital status | Married | 1678 (32.3%) | 3909 (55.9%) |  |
|  | Divorced | 106 (2.0%) | 238 (3.4%) |  |
|  | Never married | 3418 (65.7%) | 2833 (40.5%) |  |
|  | Widowed | 1 (0.0%) | 9 (0.1%) |  |
| Number of children | 1 child | n.a. | 1571 (22.5%) |  |
|  | 2-3 children |  | 2481 (35.6%) |  |
|  | four or more children |  | 115 (1.6%) |  |
|  | No children |  | 2811 (40.3%) |  |
| Physical multimorbidity | Yes | 1086 (20.6%) |  |  |
|  | No | 4180 (79.4%) |  |  |
| Regular exercise | Yes | n.a. | 5587 (79.9%) |  |
|  | No |  | 1405 (20.1%) |  |
| Weekly smoking | Yes | 1812 (34.8%) | 4945 (70.7%) |  |
|  | No | 3398 (65.2%) | 2046 (29.3%) |  |

**A3: Research question 1 for age 26 predictors**

For the first research question, we divided the sample into those who experienced a decline in mental health (i.e., an increase in psychological distress) and explored group differences based on the different social determinants of health. The main text shows the results for the predictors of age 34 in Table 1. Below, we provide the same results for the predictor variables at age 26. Among the variables, only having “physical multimorbidity” is significantly higher in the group of people with declining mental health (M=23.2% vs. 20.1%; p=0.04). All other predictors are not statistically significant. This finding aligns with later results from the main text, which identify physical multimorbidity as a main predictor of the mid-life decline in mental health.

| **Table S2.** Description of the groups of stable mental health and mental health decline at age 26 | | | | |
| --- | --- | --- | --- | --- |
|  | | | | |
| Social determinants  (all at age 26) | Share in group of **stable mental health** | Share in group of  **mental health decline** | Difference | p-value  (t-test) |
| ­Cognitive ability in  childhood (mean score) | 16.5 | 16.3 | 0.2 | 0.264 |
| **Income and employment** |  |  |  |  |
| Income quintile 5 (highest) | 17.0% | 15.1% | 1.9% | 0.16 |
| Income quintile 1 (lowest) | 15.1% | 17.4% | -2.3% | 0.09 |
| Working part-time | 7.7% | 7.1% | 0.6% | 0.50 |
| Unemployed | 3.1% | 3.1% | 0.0% | 0.95 |
| Having a disability | 2.1% | 1.8% | -0.3% | 0.489 |
| Looking after home/family | 9.3% | 11.3% | -1.9% | 0.08 |
| **Family factors** |  |  |  |  |
| Being married | 32.7% | 30.4% | 2.3% | 0.17 |
| **Physical and behavioural factors** |  |  |  |  |
| Physical multimorbidity | 20.1% | 23.2% | -3.1% | 0.04 |
| Weekly smoking | 29.0% | 30.7% | 1.8% | 0.220 |

**Notes.** Decline in mental health is defined as an increase in psychological distress on the Malaise scale by at least two points (=1 Standard Deviation) from the age of 34 to 42; all other individuals are included in the “stable mental health” group

**A4: Additional information on the RandomForest algorithm**

In this section, we provide some additional information on the Random Forest algorithm in the main analysis section. The Random Forest algorithm was used to identify the variables which are most predictive of the mental health decline in mid-life. As suggested by Fife and D’Onofrio (2023), our paper uses R package “party” and its function “cforest”. The cforest function establishes the Random Forest model using an ensemble of 1000 prediction trees (including all suggested Social Determinants of Health as possible predictors) and the function “estimates” to derive the Variable Importance for each variable. Besides the Variable Importance (VI; Root Mean Squared Error (MSE)) for each predictor, the algorithm also produces a score of overall prediction accuracy (Out of Bag accuracy of prediction) and R Squared, which are of less relevance for our research questions as our goal is variable selection rather than overall prediction accuracy and the R squared of predicting individuals’ level of change is expected to be low. As expected, the overall prediction of predicted probability was low (R^2^= 1.01%), showing that individual changes in health cannot be predicted with high accuracy. The social determinants selected therefore provide a measure of population-level trends, rather than an accurate risk prediction for each individuals. By additionally controlling for the Malaise score at age 34 throughout all analyses, we ensure that the decline is not dependent on the baseline level.

**A5: Selection of co-variates for the regression analyses performed in Table 2**

- **M1 (Social class):** This model focuses on estimating the association between of social class at birth and the mid-life decline. It only includes sex as additional control variable. All other variables temporarily lay between birth factors and mental health in mid-life, and could therefore be potential mediators of the relationship. We keep social class as control variable in the models M2 – M7.
- **M2 (Education):** For interpreting the association between education and mental health, we control for background factors of social class, sex and cognitive ability at age 10, which happened earlier in people’s lifetime. Other factors are likely become mediators for the link of education and mental health, and are therefore not included in the model.
- **M3 (Income):** Income is added to the control variables of social class, sex, cognitive ability and education, which are all likely to be confounders for the relationship between income and mental health. Other factors are likely to be mediators or competing exposures and are not included in model M3. They are however included in the models M4 and M5, where income remains statistically significant even if such potential mediators and competing exposures area added.
- **M4 (Employment status) and M5 (work conditions):** Employment status and work conditions are included in addition to previous control variables (social class, sex, cognitive ability and education). We also control for income so that effects of work conditions and employment status needs to be relevant beyond income differences.
- **M6 (Family factors) and M7 (Physical health):** We control for the background factors which happen earlier in life (social class, sex, cognitive ability and education).

**A6: Predictors of mental health decline from age 26 – 34**

We perform equivalent logistic regression analyses and predicted probabilities (as produced in Table 2 in the main text) for social class, income and physical multimorbidity on the decline in mental health from the ages of 26 – 34 (replicating model M1, M3 and M7 as S1, S3 and S7). The results of this analysis are shown in Table S3. As reported in the main text (lower part of Figure 2), the table shows that low social class at birth is not significantly associated with a decline in mental health from the age of 26 – 34 (OR = 1.03 ; CI[0.83–1.28]. On the other hand, income (OR = 0.58; [0.44 – 0.76]) and physical health (1.40;[1.19–1.65]) are significantly associated with the decline in mental health from age 26 – 34.

| **Table S3.** Logistic regression; outcome: the likelihood of experiencing a mid-life decline in mental health from age 26 to 34 (Odds Ratios) | | | |
| --- | --- | --- | --- |
|  | **S1**  **Social class** | **S3**  **Income** | **S7**  **Physical health** |
| **Social class and education** |  |  |  |
| Low social class at birth: (ref=high) | 1.03 [0.83–1.28] | 0.84 [1.15–1.72] | 0.96 [0.76–1.19] |
| Medium social class at birth: (ref=high) | 1.12 [0.94–1.33] | 1.04 [0.88–1.23] | 1.07 [0.90–1.27] |
| **Income and employment** |  |  |  |
| Income quintile 2 (ref= q1) |  | 0.98 [0.76 – 1.25] |  |
| Income quintile 3 (ref= q1) |  | 0.96 [0.76 – 1.22] |  |
| Income quintile 4 (ref= q1) |  | 0.81 [0.62 – 1.04] |  |
| Income quintile 5 (ref= q1) |  | 0.58 [0.44 – 0.76] |  |
| Looking after home/family (ref= all other) |  |  |  |
| Working parttime |  |  |  |
| **Working conditions** |  |  |  |
| Job satisfaction high (ref = low) |  |  |  |
| Job security high (ref = low) |  |  |  |
| **Family factors** |  |  |  |
| Being married at age 34 (ref= unmarried) |  |  |  |
| Having no children aged 34 (ref=1 child) |  |  |  |
| Having 2-3 children (ref=1 child) |  |  |  |
| Having four or more children |  |  |  |
| **Behavioural factors and physical health** |  |  |  |
| Having physical multimorbidity  aged 26 (ref = no) |  |  | 1.40 [1.19–1.65] |
| **Covariates** |  |  |  |
| Mental health age 26 | 0.88 [0.85–0.92] | 0.86 [0.82–0.90] | 0.86 [0.82–0.90] |
| Sex (being male) | 0.97 [0.85–0.12] | 1.04 [0.90–1.21] | 0.99 [0.86–1.13] |
| Cognitive ability aged 10 |  | 0.99 [0.97–1.00] | 0.97 [0.96–0.99] |
| Notes. 95%. CIs shown. N = 6,992 for all analyses. | | | |

**Appendix B: Supplementary statistical analyses**

Appendix B has the purpose of providing various supplementary statistical analyses on the robustness of the results displayed in the main text above. There are three main supplementary analyses performed.

Section B1 outlines the results for an alternative coding of mental health decline. Section B2 provides a LASSO regression as an alternative analytical tool. Section B3 provides more details on the handling of missing data in this paper and provides a full-cases analysis as an alternative to the main text.

**B1: Alternative coding of mental health decline**

In the main text, we defined the decline in mental health as an increase in psychological distress on the Malaise scale by at least two points (one standard deviation) between the ages of 34 to 42.

In the alternative coding, Decline in mental health is defined as being under the threshold at age 34, and declining in mental health by at least 2 points (i.e., one standard deviation) on the Malaise scale from 34 – 42. Stable mental health is defined as being under the threshold for mental distress at age 34 (Malaise score <4 and not having a decline in mental health). We repeated the main analysis shown in Table S4 based on this alternative coding. The results are shown in Table S4 below. The findings in terms of the significant factors are consistent with Table 2 in the main text.

| **Table S4.** Logistic regression; outcome: the likelihood of experiencing a mid-life decline in mental health from age 34 to 42 (Odds Ratios) | | | | | | | |
| --- | --- | --- | --- | --- | --- | --- | --- |
|  | **M1**  **Social class** | **M2**  **Education** | **M3**  **Income** | **M4**  **Employment status** | **M5**  **Work conditions** | **M6**  **Family**  **factors** | **M7**  **Physical health** |
| **Social class and education** |  |  |  |  |  |  |  |
| Low social class at birth (ref=high) | 1.52 [1.25–1.85] | 1.38 [1.12–1.71] | 1.37 [1.11–1.69] | 1.36 [1.10–1.68] | 1.35 [1.10–1.67] | 1.38 [1.12–1.70] | 1.39 [1.12–1.71] |
| Medium social class at birth (ref=high) | 1.02 [0.87–1.00] | 0.97 [0.81–1.15] | 0.97 [0.82–1.16] | 0.97 [0.82–1.15] | 0.96 [0.81–1.15] | 0.96 [0.81–1.15] | 0.97 [0.81–1.15] |
| **Income and employment** |  |  |  |  |  |  |  |
| Income quintile 2 (ref= q1) |  |  | 0.84 [0.66 – 1.07] | 0.85 [0.66 – 1.08] | 0.84 [0.66 – 1.07] |  |  |
| Income quintile 3 (ref= q1) |  |  | 0.98 [0.77 – 1.24] | 0.92 [0.72 – 1.19] | 0.98 [0.77 – 1.24] |  |  |
| Income quintile 4 (ref= q1) |  |  | 0.78 [0.61 – 1.01] | 0.73 [0.56 – 0.95] | 0.78 [0.60 – 1.00] |  |  |
| Income quintile 5 (ref= q1) |  |  | 0.68 [0.52 – 0.89] | 0.64 [0.48 – 0.85] | 0.68 [0.52 – 0.88] |  |  |
| Looking after home/family (ref= all other) |  |  |  | 1.06  [0.81 – 1.39] |  |  |  |
| Working parttime |  |  |  | 0.84 [0.66 – 1.05] |  |  |  |
| **Working conditions** |  |  |  |  |  |  |  |
| Job satisfaction high (ref = low) |  |  |  |  | 0.80 [0.61 – 1.05] |  |  |
| Job security high (ref = low) |  |  |  |  | 0.91 [0.69 – 1.22] |  |  |
| **Family factors** |  |  |  |  |  |  |  |
| Being married at age 34 (ref= unmarried) |  |  |  |  |  | 0.91 [0.64 – 1.32] |  |
| Having no children aged 34 (ref=1 child) |  |  |  |  |  | 0.92 [0.77 – 1.11] |  |
| Having 2-3 children (ref=1 child) |  |  |  |  |  | 0.96 [0.80 – 1.14] |  |
| Having four or more children |  |  |  |  |  | 1.05 [0.60 – 1.74] |  |
| **Behavioural factors and physical health** |  |  |  |  |  |  |  |
| Having physical multimorbidity  aged 26 (ref = no) |  |  |  |  |  |  | 1.28 [1.07–1.54] |
| **Covariates** |  |  |  |  |  |  |  |
| Mental health age 34 | 0.98 [0.92–1.05] | 0.97 [0.91–1.04] | 0.97 [0.91–1.03] | 0.97 [0.91–1.03] | 0.96 [0.90–1.03] | 0.97 [0.91–1.03] | 0.97 [0.91–1.03] |
| Sex (being male) | 0.87 [0.77–1.00] | 0.86 [0.76–0.99] | 0.92 [0.80–1.06] | 0.89 [0.76–1.05] | 0.97 [0.84–1.12] | 0.87 [0.76–0.99] | 0.88 [0.77–1.00] |
| University education aged 34  (ref= low education) |  | 0.88 [0.75–1.02] | 0.96 [0.82–1.13] | 0.97 [0.82–1.15] | 0.96 [0.82–1.14] | 0.88 [0.76–1.03] | 0.88 [0.75–1.03] |
| Cognitive ability aged 10 |  | 0.99 [0.97–1.00] | 0.99 [0.98–1.01] | 0.99 [0.98–1.01] | 0.99 [0.98–1.01] | 0.99 [0.98–1.00] | 0.99 [0.97–1.00] |
| Notes. 95% Cis shown. N = 6,992 for all analyses. | | | | | | | |

**B2: LASSO Regression as alternative analysis**

To verify the results acquired from the Random Forest, we also computed a LASSO regression suing the R package “caret”. The results are shown in Figure S3. The results show a very similar list of predictors compared to Figure 2 in the main text. “Low social class” and “income at age 34” are also among the top 3 predictors as the RandomForest in the main text. Physical multimorbidity, ranks at place 2 in the LASSO regression, and is also one of the key predictors identified in the main text which remains statistical significant in the main logistic regression analysis (see Table 2 in the main text). Overall, all variables which play a key role in the main text and stay statistically significant throughout all analyses are also detected by this alternative Machine Learning technique.

**(Insert Figure S1 here)**

**B3: Handling of missing values**

The full sample consists of all participants who provided a valid answer to question on mental distress at ages 34 and 42 (N= 6992). Before conducting step 2 and 3 of the analysis, missing values in all explanatory variables were imputed. To do so, we used the R package RandomForest, which predictor missing values with adequate error rates in prediction (NRMSE = 0.35 for imputing quantitative and PFC= 0.116 for
categorical variables). This approach allows to be consistent between analysis step 2 and 3. Finally, we also
conducted a full-case analysis without the imputation of missing values. The findings are shown in Table S5
and remain stable in terms of statistical significance as shown in Table 2 of the main text.

| **Table S5.** Full-case analysis; Logistic regression; outcome: the likelihood of experiencing a mid-life decline in mental health from age 34 to 42 (Odds Ratios) | | | | | | | |
| --- | --- | --- | --- | --- | --- | --- | --- |
|  | **M1**  **Social class** | **M2**  **Education** | **M3**  **Income** | **M4**  **Employment status** | **M5**  **Work conditions** | **M6**  **Family**  **factors** | **M7**  **Physical health** |
| **Social class and education** |  |  |  |  |  |  |  |
| Low social class at birth (ref=high)s | 1.46 [1.20–1.78] | 1.38 [1.10–1.74] | 1.37 [1.09–1.72] | 1.37 [1.09–1.72] | 1.35 [1.10–1.70] | 1.38 [1.10–1.73] | 1.47 [1.13–1.92] |
| Medium social class at birth (ref=high) | 1.02 [0.88–1.22] | 0.99 [0.82–1.20] | 0.99 [0.82–1.20] | 0.99 [0.82–1.20] | 0.98 [0.81–1.19] | 0.99 [0.82–1.19] | 1.04 [0.84–1.30] |
| **Income and employment** |  |  |  |  |  |  |  |
| Income quintile 2 (ref= q1) |  |  | 0.96 [0.73 – 1.24] | 0.96 [0.74 – 1.25] | 0.97 [0.75 – 1.27] |  |  |
| Income quintile 3 (ref= q1) |  |  | 1.12 [0.86 – 1.46] | 1.07 [0.82 – 1.41] | 1.14 [0.88 – 1.48] |  |  |
| Income quintile 4 (ref= q1) |  |  | 0.85 [0.64 – 1.12] | 0.81 [0.61 – 1.09] | 0.86 [0.65 – 1.13] |  |  |
| Income quintile 5 (ref= q1) |  |  | 0.70 [0.52 – 0.94] | 0.67 [0.49 – 0.91] | 0.70 [0.52 – 0.95] |  |  |
| Looking after home/family (ref= all other) |  |  |  | 1.00  [0.75 – 1.34] |  |  |  |
| Working parttime |  |  |  | 0.87 [0.68 – 1.12] |  |  |  |
| **Working conditions** |  |  |  |  |  |  |  |
| Job satisfaction high (ref = low) |  |  |  |  | 0.84 [0.63 – 1.13] |  |  |
| Job security high (ref = low) |  |  |  |  | 0.94 [0.69 – 1.29] |  |  |
| **Family factors** |  |  |  |  |  |  |  |
| Being married at age 34 (ref= unmarried) |  |  |  |  |  | 0.97 [0.65 – 1.51] |  |
| Having no children aged 34 (ref=1 child) |  |  |  |  |  | 0.91 [0.75 – 1.11] |  |
| Having 2-3 children (ref=1 child) |  |  |  |  |  | 0.92 [0.76 – 1.12] |  |
| Having four or more children |  |  |  |  |  | 1.09 [0.75 – 1.11] |  |
| **Behavioural factors and physical health** |  |  |  |  |  |  |  |
| Having physical multimorbidity  aged 26 (ref = no) |  |  |  |  |  |  | 1.25 [1.02–1.52] |
| **Covariates** |  |  |  |  |  |  |  |
| Mental health age 34 | 0.88 [0.85–0.92] | 0.86 [0.82–0.90] | 0.85 [0.82–0.89] | 0.85 [0.81–0.89] | 0.85 [0.81–0.89] | 0.85 [0.82–0.89] | 0.84 [0.79–0.88] |
| Sex (being male) | 0.87 [0.77–0.99] | 0.84 [0.72–0.97] | 0.90 [0.77–1.72] | 0.87 [0.74–1.04] | 0.94 [0.80–1.11] | 0.83 [0.72–0.97] | 0.86 [0.73–1.02] |
| University education aged 34  (ref= low education) |  | 0.96 [0.82–1.14] | 1.05 [0.88–1.26] | 1.06 [0.89–1.26] | 1.05 [0.88–1.25] | 0.97 [0.82–1.15] | 1.07 [0.88–1.29] |
| Cognitive ability aged 10 |  | 0.99 [0.98–1.01] | 0.99 [0.98–1.01] | 0.99 [0.98–1.01] | 0.99 [0.98–1.01] | 0.99 [0.98–1.01] | 0.99 [0.98–1.01] |
| N | 6512 | 5209 | 5209 | 5208 | 5196 | 5195 | 3946 |
| Notes. 95% Cis shown. | | | | | | | |
